# Editorial: Unravelling Citrus Huanglongbing Disease

**DOI:** 10.3389/fpls.2020.609655

**Published:** 2020-11-13

**Authors:** Rhuanito Soranz Ferrarezi, Christopher Isaac Vincent, Alberto Urbaneja, Marcos Antonio Machado

**Affiliations:** ^1^Indian River Research and Education Center, Institute of Food and Agricultural Sciences, University of Florida, Fort Pierce, FL, United States; ^2^Citrus Research and Education Center, Institute of Food and Agricultural Sciences, University of Florida, Lake Alfred, FL, United States; ^3^Unidad de Entomología, Centro de Protección Vegetal y Biotecnología, Instituto Valenciano de Investigaciones Agrarias, Moncada, Spain; ^4^Agronomic Institute, Campinas, Brazil

**Keywords:** *Candidatus* Liberibacter spp., disease management, infection, citriculture, vector management, citrus under protected screen

Huanglongbing (HLB) or citrus greening is a disease caused by the unculturable, fastidious, phloem-restrictive, Gram-negative bacterium *Candidatus* Liberibacter spp. Currently, there are three species linked to the disease. The Asian form associated with *Candidatus* Liberibacter asiaticus (*C*Las) is heat-tolerant and can survive well above 30°C. The African (*Candidatus* Liberibacter africanus) and American forms are heat-sensitive and develop between 22 and 25°C (*Candidatus* Liberibacter americanus) (Bové, 2006). Huanglongbing is vector-transmitted mainly by the African citrus psyllid *Trioza erytreae* Del Guercio (Hemiptera: Triozidae) and the Asian citrus psyllid (ACP) *Diaphorina citri* Kuwayama (Hemiptera: Psyllidae), with two other psyllids also reported as vectors, *D. communis* Mathur and *Cacopsilla citrisuga* (Yang & Li) (Hemiptera: Pysllidae). The disease was first described in 1929 and reported in China in 1943. The African variation was reported in South Africa in 1947. The disease was reported in Brazil (São Paulo) in 2004 and the United States (Florida) in 2005. More than 20% of citrus trees in Brazil and 90% in Florida are currently affected, with symptomatic trees present in Texas and California. Huanglongbing is present and affects several citrus-producing countries of Asia, sub-Saharan Africa, and America (except for Bolivia, Chile, Perú, and Uruguay). The Mediterranean Basin and Australia are still free of HLB. The threat to HLB-free countries is constant due to the proximity of the disease and its vectors and the unstoppable increase in international trade.

*Candidatus* Liberibacter asiaticus can infect most citrus species, cultivars, and hybrids. Leaves of infected trees develop a blotchy mottle symptom (yellowing vein and an asymmetrical chlorosis). Infected branches suffer substantial leaf drop, resulting in severe canopy thinning. Fibrous root density decreases nearly 30%, directly affecting water and nutrient uptake, severely reducing fruit yield, and demanding more frequent irrigation and improved mineral nutrition practices. Fruit from HLB-affected trees are often lopsided, poorly colored, and contain aborted seeds, with low commercial value due to small size and quality. The juice from affected fruit present low soluble solids content, high acidity, and bitterness.

There is no cure for the disease yet. Current management strategies focus on either delaying infection or managing infected trees. Methods of delaying infection include removal of symptomatic trees, planting and resetting using HLB-free nursery trees, protection of grove edges and intensive monitoring and control of the vectors mainly using physical, chemical, and biological control methods. Management of infected trees includes adjusting soil pH, enhancing nutritional programs, and improving irrigation management based on altered tree capacities and needs when affected by HLB. Research has evolved rapidly to address this devastating challenging, and several recent alternatives based on psyllid management, bactericides, cultural practices (thermotherapy and vector exclusion using netting), and genetic transformation have been tested. While most attempts at management have focused on a single component of the disease pyramid, most do not explicitly consider multiple elements at the same time.

This Research Topic is a collection of 9 articles from 49 co-authors and present the latest advances in managing the HLB pathosystem, focusing on assessments of near-term feasible practices in the context of the vector, pathogen, host plant, and environment.

## Vector

Britt et al. identified possible biological control candidates by conducting one of the most comprehensive surveys of natural ACP populations in major citrus production regions spanning 21 counties in Florida. By optimizing PCRs and RT-PCRs, authors successfully detected and monitored the prevalence of five previously identified ACP-associated RNA and DNA viruses throughout Florida citrus groves, which include: *D. citri*-associated C virus, *D. citri* flavi-like virus, *D. citri* densovirus, *D. citri* reovirus, and *D. citri* picorna-like virus. High-throughput sequencing generated data also revealed that the most abundant virus in Florida ACP populations was citrus tristeza virus (CTV), which is not an ACP-associated virus, suggesting persistent presence of CTV infection in citrus throughout Florida groves. Collectively, information obtained from the study may help guide the direction of biotechnological pest control efforts involving several viruses that were detected for the first time in Florida ACP populations, including two newly identified ACP-associated viruses.

Khan et al. evaluated the brown lacewing *Sympherobius barberi* as a biological control agent against the *D. citri* and frozen eggs of the Mediterranean flour moth *Ephestia kuehniella*. Adult *S. barberi* successfully fed on *D. citri* eggs and nymphs under both light and dark conditions. The Asian Citrus Psyllid was also suitable for the development and reproduction of *S. barberi* except for slightly prolonged larval development compared with *E. kuehniella* diet. The egg hatch from the total number of eggs laid on *D. citri* and *E. kuehniella* diets averaged 65 and 52%, respectively. Females laid 64% eggs on dimpled white paper compared to 36% combined on plain paper and leaves of citrus, orange jasmine, eggplant and cantaloupe. *Sympherobius barberi* released at densities of 2–6 adults against eggs and nymphs of *D. citri* on infested orange jasmine in cages provided a reduction of 43–81% in the number of eggs or nymphs. In the field tests on *D. citri* infested citrus trees, there was a 35% reduction in five cohorts in which developing colonies of 28–32 nymphs were provided to one *S. barberi* per cage. The findings suggest the potential of *S. barberi* as a predator of *D. citri* and to contribute to reducing HLB.

Patt et al. determined whether synthetic compounds, which were ligands of *D. citri* olfactory binding protein DCSAP4, influenced the settling and aggregation levels of psyllids on young citrus shoots. Authors found the test ligands themselves did not attract the psyllids but rather modulated the psyllid's response to a mixture of citrus volatiles. The results suggest that synthetic ligands of *D. citri* chemosensory binding proteins can be used to increase the effectiveness of citrus scent lures used to attract psyllids to monitoring traps and attract and kill devices.

## Pathogen

Vincent et al. assessed two grower-used therapies, heat treatment and foliar anti-bacterial application, following an industry claim that heat treatment improved subsequent systemic uptake of foliar-applied anti-bacterial compounds. Heat treatment and defoliation treatments reduced growth but did not affect systemic delivery of oxytetracycline. Oxytetracycline was detected in nearly all covered leaf samples in both repetitions, though at lower concentrations than in directly applied leaves. Authors concluded that neither heat treatment nor leaf age strongly affect systemic oxytetracycline delivery, and further discussed implications of the study for leaf age effects on foliar delivery and for phloem delivery of foreign compounds through foliar application.

Zhang et al. implemented an integrated strategy that includes chemotherapy, thermotherapy, and additional nutrition treatment in three different field trials over 3 consecutive years. Analysis showed that most of the tested chemicals were effective to some degree in killing or suppressing the bacterium, with higher therapeutic efficacy. Trunk-injected penicillin G potassium was the most effective chemical treatment in all groves, followed by oxytetracycline, and silver nitrate delivered as foliar sprays. Although the steam heat treatment and additional nutrition did not eliminate or suppress HLB over the long term, these treatments did positively affect tree growth and recovery in the short term. Overall, the results provided new insights into HLB control method and strategy for integrated management for HLB epidemic groves.

## Host Plant

Deng et al. examined transverse sections of leaf lamina and midribs with light and epifluorescence microscopy to determine anatomical characteristics that underlie HLB-tolerant mechanisms operating among “Bearss” lemon, “LB8-9” Sugar Belle® mandarin and its sibling trees compared with HLB-sensitive “Valencia” sweet orange. Although there were physical, morphological, and pathological similarities in the examined foliage, internal structural preservation in lemon and mandarin was superior compared with HLB-sensitive sweet orange and siblings of mandarin. Intriguingly, there was substantial phloem regeneration in the tolerant types that may compensate for the dysfunctional phloem, in comparison with the sensitive selections. Authors attribute the lower levels of phloem disruption and greater phloem regeneration as two key elements that contribute to HLB tolerance in diverse citrus cultivars.

Huang et al. genotyped an intergeneric F1 population of sweet orange and trifoliate orange by genotyping-by-sequencing and constructed high-density SNP-based genetic maps separately for trifoliate orange and sweet orange. Progenies of the F1 population and their parents were planted in a replicated field trial, exposed to intense HLB pressure for 3 years, and then evaluated for susceptibility to HLB over 2 years. Most of the identified QTLs each explained 18–30% of the phenotypic variation, indicating their major role in determining HLB responses. These results show, for the first time, a quantitative genetic nature as well as the presence of major loci for the HLB tolerance in trifoliate orange. The results suggest that sweet orange also contains useful genetic factors for improving HLB tolerance in commercial citrus varieties. Findings are valuable and timely for genetic solutions to the devastating HLB crisis through breeding, genetic engineering, or genome editing.

## Environment

Ferrarezi et al. assessed the ability of large-scale enclosed screenhouses to exclude the ACP, stop HLB inoculation and dissemination, and improve fruit yield of in-ground and container-grown 6-year-old “Ray Ruby” grapefruit at super-high planting densities relative to open-air trees. Despite the weather-related damages to the screens, only trees cultivated in open-air tested positive for *C*Las after 6 years. There was fast disease progression for all outside treatments, with 100% infection. The screenhouses provided disease exclusion, increased fruit yield and fruit quality, representing an alternative for growers interested in producing high-quality fruit for the fresh market.

Dala-Paula et al. provided a review analyzing and discussing the effects of HLB on orange juice quality to help the citrus industry manage the quality of orange juice and guide future research needs. Symptomatic oranges show higher titratable acidity and lower soluble solids, solids/acids ratio, total sugars, and malic acid levels. Among flavor volatiles, ethyl butanoate, valencene, decanal and other ethyl esters are lower, but many monoterpenes are higher in symptomatic fruit compared to healthy and asymptomatic fruit. The disease also causes an increase in secondary metabolites in the orange peel and pulp, including hydroxycinnamic acids, limonin, nomilin, narirutin, and hesperidin. Resulting from these chemical changes, juice made from symptomatic fruit is described as distinctly bitter, sour, salty/umami, metallic or musty and lacking in sweetness and fruity/orange flavor. Those effects are reported in both “Valencia” and “Hamlin” sweet oranges, two cultivars that are commercially processed for juice in Florida. Earlier research showed that HLB-induced off-flavor was not detectable in juice made with up to 25% symptomatic fruit in healthy juice, by chemical or sensory analysis. However, a blend with a higher proportion of symptomatic juice would present a detectable and recognizable off flavor.

## Outlook and Future Challenges

Huanglongbing will continue to be the main citrus disease in the world. In recent years, there has been a significant increase in the volume of scientific information on all aspects of citrus relations with the bacteria, the vector, and the environment. Despite all this information, the scientific community has not yet managed to bring a definitive solution to the control of the disease. The disease is highly complex, affecting the transport system leading to phloem transport and carbohydrate distribution dysfunction.

The special edition of Frontiers dedicated to HLB was an attempt to expand the forum for the presentation of new results and to contribute with new approaches to the understanding of the disease and its control. Publishers hope that these opportunities will continue and expand. Only the contribution of the scientific and technological community can bring a definitive solution to the main challenges of citrus production worldwide.

## Author Contributions

All authors listed have made a substantial, direct and intellectual contribution to the work, and approved it for publication.

## Conflict of Interest

The authors declare that the research was conducted in the absence of any commercial or financial relationships that could be construed as a potential conflict of interest.
